# MSCs and their exosomes: a rapidly evolving approach in the context of cutaneous wounds therapy

**DOI:** 10.1186/s13287-021-02662-6

**Published:** 2021-12-04

**Authors:** Faroogh Marofi, Kozlitina Iuliia Alexandrovna, Ria Margiana, Mahta Bahramali, Wanich Suksatan, Walid Kamal Abdelbasset, Supat Chupradit, Maryam Nasimi, Marwah Suliman Maashi

**Affiliations:** 1 Immunology Research Center (IRC), Tabriz University of Medical Sciences, Tabriz, Iran; 2 Sechenov First Moscow State Medical University, Moscow, Russian Federation; 3 Department of Anatomy, Faculty of Medicine, Universitas Indonesia, Jakarta, Indonesia; 4 Master’s Programme Biomedical Sciences, Faculty of Medicine, Universitas Indonesia, Jakarta, Indonesia; 5 Biotechnology Department, University of Tehran, Tehran, Iran; 6 Faculty of Nursing, HRH Princess Chulabhorn College of Medical Science, Chulabhorn Royal Academy, Bangkok, 10210 Thailand; 7 Department of Health and Rehabilitation Sciences, College of Applied Medical Sciences, Prince Sattam Bin Abdulaziz University, Al Kharj, Saudi Arabia; 8 Department of Physical Therapy, Kasr Al-Aini Hospital, Cairo University, Giza, Egypt; 9 Department of Occupational Therapy, Faculty of Associated Medical Sciences, Chiang Mai University, Chiang Mai, 50200 Thailand; 10 Tehran University of Medical Sciences, Tehran, Iran; 11 Stem Cells and Regenerative Medicine Unit at King Fahad Medical Research Centre, Jeddah, Saudi Arabia

**Keywords:** Mesenchymal stem/stromal stem cell (MSC), Cutaneous wounds, Exosome, Paracrine factors, Differentiation

## Abstract

Currently, mesenchymal stem/stromal stem cell (MSC) therapy has become a promising option for accelerating cutaneous wound healing. In vivo reports have outlined the robust competences of MSCs to offer a solid milieu by inhibition of inflammatory reactions, which in turn, enables skin regeneration. Further, due to their great potential to stimulate angiogenesis and also facilitate matrix remodeling, MSCs hold substantial potential as future therapeutic strategies in this context. The MSCs-induced wound healing is thought to mainly rely on the secretion of a myriad of paracrine factors in addition to their direct differentiation to skin-resident cells. Besides, MSCs-derived exosomes as nanoscale and closed membrane vesicles have recently been suggested as an effective and cell-free approach to support skin regeneration, circumventing the concerns respecting direct application of MSCs. The MSCs-derived exosomes comprise molecular components including lipid, proteins, DNA, microRNA, and also mRNA, which target molecular pathways and also biological activities in recipient cells (e.g., endothelial cell, keratinocyte, and fibroblast). The secreted exosome modifies macrophage activation, stimulates angiogenesis, and instigates keratinocytes and dermal fibroblast proliferations as well as migrations concurrently regulate inherent potential of myofibroblast for adjustment of turnover of the ECM. In the present review, we will focus on the recent findings concerning the application of MSCs and their derivative exosome to support wound healing and skin regeneration, with special focus on last decade in vivo reports.

## Introduction

Because of the high rates of the incidence of skin damages resulting from acute or chronic wounds including extensive burns, trauma, and ulcers of various etiologies, recognizing or designing more efficient strategies to support cutaneous regeneration and ameliorate damaged skin’s normal functions is urgently requisite [1, 2]. Skin wound healing at the wound tissue comprises some complicated and dynamic procedures involving several cell types, growth factors, extracellular matrix (ECM), and also blood vessels [3, 4]. Advancements in therapeutic options for accelerating cutaneous wound healing have been potentiated by application of mesenchymal stem/stromal cells (MSCs)-based treatments [5, 6]. As skin-resident MSCs in normal skin contribute largely to wound healing, employing exogenous MSCs seems to be a rational option to restore wound skin [7]. In addition to the differentiation into skin cells (e.g., keratinocytes and myofibroblasts), MSCs mainly secrete a myriad of soluble mediators such as vascular endothelial growth factor (VEGF), fibroblast growth factors (FGFs), hepatocyte growth factor, transforming growth factor (TGF-β), interleukin (IL)-4, IL-10, nitric oxide (NO), and prostaglandin E2 (PGE2) [7, 8]. These mediators in turn mainly suppress the immune response, induce angiogenesis, regulate fibroblast and keratinocyte biological functions, and also support anti-fibrotic effects in wound tissue [9]. Nonetheless, substantial inconsistencies in the delivery protocols, and also diversities among MSCs populations obstruct their utility in the clinic. Irrespective of some unclear reports, MSC's direct differentiation into phenotypes typical of resident skin cells during cutaneous wound healing has not been completely validated [10]. These challenges and controversies have outlined the significance of using MSCs secretome, such as MSCs-derived exosome, to ameliorate cutaneous wounds due to the existence of soluble factors at high levels.

During recent years, MSCs-exosomes have attracted increasing attention as an innovative cell-free approach in the context of wound healing, circumventing the concerns related to the MSC's direct application [11]. The MSCs-exosomes include frequent cytoplasmic and membrane proteins, comprising receptors, enzymes, transcription factors, lipids, ECM proteins, and also nucleic acids, including mitochondrial DNA (mtDNA), single-stranded DNA (ssDNA), double-stranded DNA (dsDNA), messenger RNA (mRNA), and microRNA (miRNA) [12]. These exosomes can generally affect biological events such as proliferation, migration, apoptosis, and also immunomodulatory reactions in recipient cells by conveying their contents, and thereby enable modifying regenerative programs of target organs through targeting central signaling axes, such as phosphoinositide 3-kinases (PI3Ks) /AKT, Janus kinase (JAK)/signal transducer and activator of transcription (STAT), transforming growth factor β (TGF-β)/Smad and Wnt/β-catenin pathways [13–15]. Indeed, MSCs-exosome regulates macrophage activation, triggers angiogenesis, begins keratinocytes and dermal fibroblast proliferation and migration, and also adjust myofibroblast's inherent capacities to modify the turnover of the ECM [16]. These reports have evidenced that exosomes promote the biological attributes of keratinocytes, fibroblasts, immune cells, and endothelial cells, thereby offering a reliable therapeutic plan for skin recovery [17].

Herein, we will emphasize the therapeutic potential of MSCs and also their exosomes to accelerate cutaneous chronic wounds, and also deliver an overview respecting the biological functions affected by MSCs and their exosomes to support wound amelioration and skin regeneration.

## Wound healing phases

Cutaneous wound healing is a critical physiological procedure comprising the collaboration of numerous cell types and their secreted molecules [18, 19]. Comprehensive discussion respecting the cellular and molecular events complicated in the wound healing process is beyond the scope of this study; thereby readers are referred to some informative reviews in this regard [20–22]. In this section, a brief overview concerning the wound healing process and responding phases has been provided.

Cell and biochemical proceedings in wound healing mainly can be separated into the four main stages including the hemostasis phase, inflammatory phase, proliferative phase, and maturation phases also called the remodeling [23, 24]. Hemostasis as the primary stage of healing instigates immediately after the injury to halt the bleeding. Throughout this phase, platelets' interactions with collagen results eventually in platelets activation and resultant aggregation [25, 26]. Thrombin as the central factor stimulates the creation of a fibrin mesh, which in turn, reinforces platelet clumps into a stable clot. The inflammatory phase, as the second step of wound healing, concentrates on deteriorating bacteria and eliminating debris, thereby providing the wound bed for the establishment of new tissue [27, 28]. Immune cells, such as neutrophils and macrophages, play a central role in this process, and both controls bleeding and hinder infection through direct activities or secretion of a spectrum of soluble mediators [29]. The proliferative step of wound healing includes filling the wound, contraction of the wound margins by myofibroblasts activation, and also covering the wound called also epithelialization [30, 31]. Importantly, the construction of a new complex of blood vessels is robustly needed to offer sufficient oxygen and nutrients to newly established granulation tissue [32]. In the maturation phase, slow resolution of the inflammatory phase, collagen deposition, and entire coverage of the damaged site by the new tissues and finally creation of scar tissue is displayed [33]. Established tissue gradually attains strength and flexibility, and collagen is remodeled from type III to type I, and the wound completely closes [34].

Failure to advance in the steps of wound healing usually leads to chronic wounds. Venous disease, infection, diabetes, and metabolic deficiencies are common causes of the occurrence and development of cutaneous wounds [35].

## MSCs and wound healing

MSCs as well-known progenitor cells of mesodermal origin were primarily procured from bone marrow (BM) by Fridestein et al. in the 1970s [36]. They characterized MSCs by their inherent competence to adhere to tissue culture surfaces (e.g., plastic) [36]. Irrespective of the inherent potential to derive colonies from single cells (“colony forming units-fibroblastic,” CFUs-F), MSCs exhibited the capability to differentiate into adipocytes, chondrocytes, and osteocytes [36]. After that, MSCs were successfully isolated from adipose tissue, endometrium, and dermal skin tissue, and also from embryonic and fetal sources like the amniotic, umbilical cord (UC), and umbilical cord blood (UCB)/Wharton's jelly (WJ) [37–39].

Endogenous cutaneous MSCs comprise dermal papilla cells (DPC) located at the base of the hair follicle, and also the dermal sheath cells (DSC) surrounded hair follicle units. DPCs are chiefly contributed to the modulating hair follicle cycling [40, 41], whereas the DSCs are most probably complicated in substituting the dermis in response to wound via differentiation into wound healing fibroblasts [42]. Also, further dermal MSCs seem to be positioned in the interfollicular dermis [43, 44]. As well, cutaneous wounds may induce activation and recruitment of adipose-derived mesenchymal stem/stromal cells (Ad-MSCs) to the wound tissue [45]. In association with fibroblasts, mature and precursor adipocytes populate the injured zone throughout the proliferative phase of wound healing. Interestingly, compromised wound healing of lipoatrophic mice signifies that efficient recruitment of fibroblast and dermal reconstruction may arise from Ad-MSCs activities [46]. As well, stromal vascular fraction (SVF) derived from adipose tissues shows great regenerative capacities for amelioration of diabetic foot ulcers, and also soft tissue defects. SVF induces migration of fibroblasts and angiogenesis by regulation of ECM in the skin wound healing process [47]. Studies have also revealed that the epithelialization growth factor, such as epidermal growth factor (EGF), chemokines, stromal cell-derived factor (SDF-1 or CXCL12), neutrophil-activating protein-2 (NAP-2 or CXCL7), chemokine receptors (CXCR1, CCR2, and CCR3), and wound healing genes were mainly up-regulated in SVF compared with Ad-MSCs [48, 49]. Thereby, it seems that SVF implantation accelerates wound closure and improves cellularity and re-epithelialization as shown in mice models. Besides, the BM-MSCs have been implicated in cutaneous wound healing and are described to be recruited to injured tissue in early inflammation and maintained in the reconstructed dermal tissue [50].

**Fig. 1:**
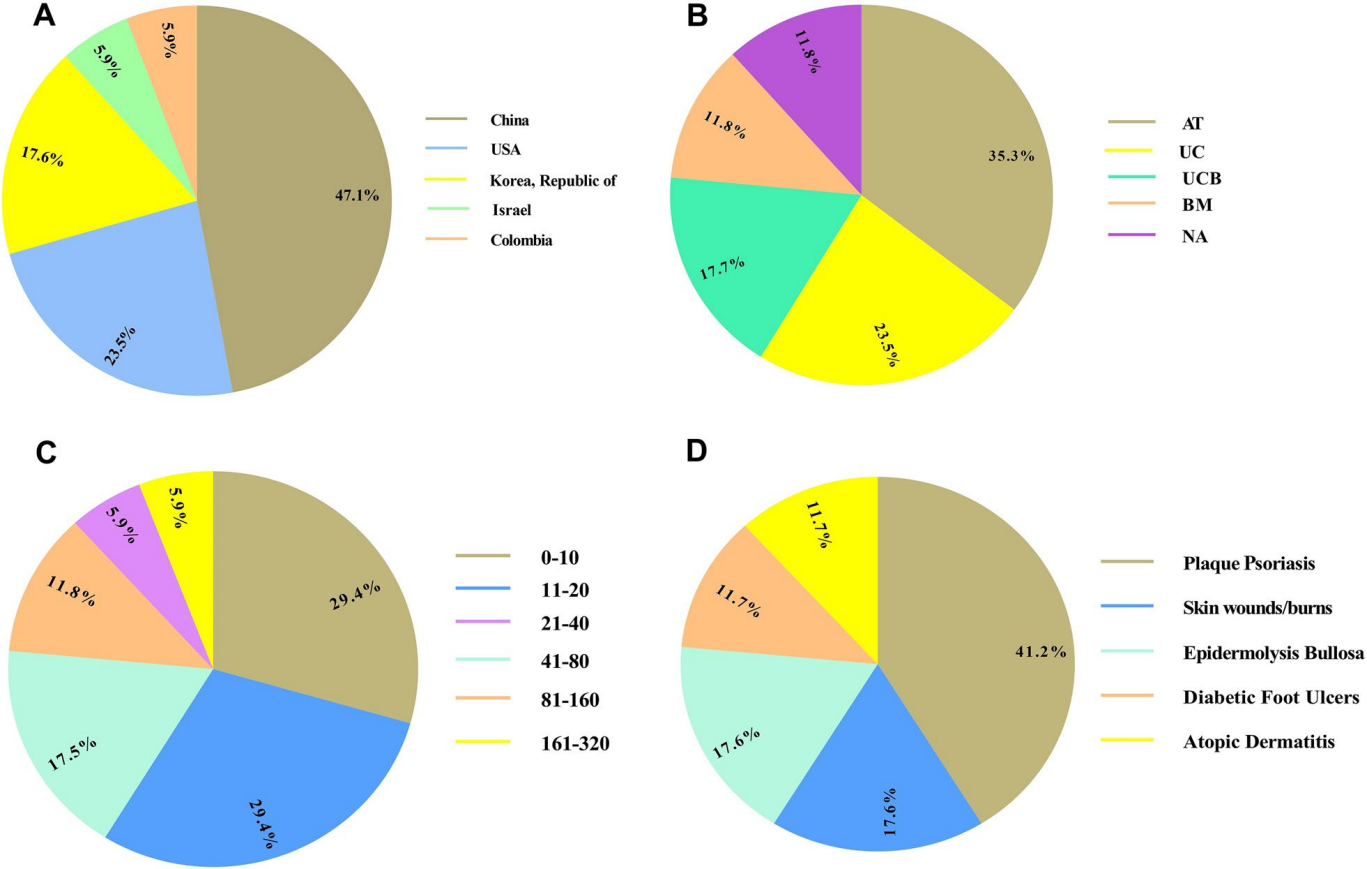
Clinical trials based on mesenchymal stem/stromal cells (MSCs) administration for accelerating cutaneous wound healing registered in ClinicalTrials.gov (August 2021). The schematic presents clinical trials respecting the MSCs therapy for participants suffering from skin ulcers depending on the study location (**A**), cell source (**B**), participant number (**C**), and condition (**D**)

Currently, due to their physiological therapeutic actions, MSCs have been exogenously employed to wounds to ameliorate both wound healing and scarring (Tables 1 and 2) (Fig. 1) [8, 51, 52]. Indeed, MSC’s differentiation potential concomitant with their unique other competencies, such as the producing immunomodulatory and pro-angiogenic soluble factors, make them a rational and rapidly developing attractive therapeutic strategy in regenerative medicine for acute and chronic wounds treatments. In this section, we deliver a concise summary concerning the MSCs therapy for wound healing in vivo. In the other sections, a comprehensive outline respecting the therapeutic benefits of MSCs application along with the potential underlying mechanisms has been offered.

**Table 1:** Mesenchymal stem/stromal cell (MSC)-based therapies for cutaneous wound healing

Cell source	Model	Results	References
BMMNC	In vitro	Verifying the wound healing capabilities of CD271 + MSCs	[193]
AT	In vivo	Facilitating the wound healing MSCs through the TLR4-dependent shaping of the wound site	[194]
BM	In vivo	Induction of the skin recovery by MSCs through the inhibition of inflammation and also enhancing the skin regeneration-related growth factors	[60]
AT	In vivo	Inhibition of the TNF-α-dependent inflammation, enhancing the anti-inflammatory M2 macrophage quantity, and stimulating TGF-β1-mediated angiogenesis, myofibroblast differentiation, and granulation tissue establishment by ppAAc delivered MSCs	[51]
BM	In vivo	Lower immunogenicity and higher infiltration of allogeneic BM-MSCs than allogeneic fibroblasts	[188]
BM	In vivo	Promoting the regeneration of DEB wounds by MSCs by the formation of functional immature anchoring fibrils	[54]
BM	In vivo	Showing the higher capacity to induce wound healing in diabetic mice by BM-MSCs than fibroblasts	[53]
BM	In vivo	Verifying the MSCs recruitment into wound skin and stimulating wound healing by transdifferentiation into several cell types	[195]
BM	In vivo	Promotion of MSCs differentiation ability and diabetic wound healing in diabetic mice by implantation of PEGylated graphene oxide-mediated quercetin-modified collagen hybrid scaffold loaded with MSCs	[58]
BM	In vivo	Promoting the viability and activity of both ISCs and MSCs by their coencapsulation supporting better wound healing	[196]
WJ	In vivo	Amelioration of the proliferation, angiogenesis, and wound healing ability of WJ-MSCs by hyperbaric oxygen in diabetic mice	[57]
UCB	In vivo	Confirming the MSCs differentiation into keratinocyte in the wound tissue	[8]
BFP	In vivo	Inducing wound healing by curcumin-loaded electrospun nanofibers along with MSCs as a bioactive dressing	[197]
BM	In vivo	Stimulating diabetic wound healing by BM-MSCs delivery using N-carboxyethyl chitosan (N-chitosan), adipic acid dihydrazide (ADH), and hyaluronic acid-aldehyde (HA-ALD) hydrogel	[59]
NA	In vivo	Inhibition of wound healing process by miR-27b du to the inhibition of MSCs migration to burned margins	[198]
BM	In vitro	Signifying the critical role of the ERK pathway in the phenotype shift of MSCs into human sweat gland cells (SGCs)	[199]
BM	In vivo	Facilitating wound healing in acute full-thickness skin wounds by collagen loaded with MSCs	[200]
BM	In vivo	Verifying the positive effect of autophagy in MSC-mediated vascularization in cutaneous wound healing by adjusting the VEGF producing	[201]
BMMNC	In vitro	Inducing the migration of skin and wound fibroblast by MSCs	[202]
PB	In vivo	Improving the wound healing sheep skin through promoting the expression of hair-keratin (hKER) and Collagen1 gene (Col1α1) by MSCs	[203]
AT	In vivo	Amelioration of diabetic wounds by decellularized silk fibroin scaffold primed with MSCs	[204]
BM	In vitro	Improving the expression of ICAM-1 in MSCs leading to the promotion of their migration by TNF-α	[205]
BMMNC	In vivo	Amelioration of wound damages by MSCs-expressing angiopoietin-1 gene	[130]
BM	In vivo	Promoting the functions of MSCs in wound bed by their pretreatment with TGF-β1	[206]
AT	In vivo	Improving the wound healing rate in diabetic rats without any enhancement in volume density of the vessels and collagen fibers by MSCs	[207]

Bone marrow-derived mononuclear cells (BMMNCs), Adipose tissue (AT), Bone marrow (BM), Umbilical cord blood (UCB), Wharton's jelly (WJ), Buccal fat pad (BFP), Toll-like receptor 4 (TLR4), Tumor necrosis factor α (TNFα), Transforming growth factor-beta (TGF-β), Dystrophic epidermolysis bullosa (DEB), Insulin secreting cells (ISCs), Extracellular signal-regulated kinase (ERK), Vascular endothelial growth factor (VEGF), Intercellular adhesion molecule-1 (ICAM-1), MicroRNAs (miRNAs)

**Table 2:** A summary of clinical trials based on mesenchymal stem/stromal cell therapies for accelerating cutaneous wound healing registered in ClinicalTrails.gov (August 2021)

Condition	Cell source	Phase	Participant number	Status	Location	NCT number
Skin Wound Injury	UC	1	20	Completed	China	NCT02669199
Skin Ulcers	UC	1	20	Completed	China	NCT02685722
Burn Wound	NA	1	15	Completed	USA	NCT02104713
Plaque Psoriasis	UC	1/2	30	Unknown	China	NCT02491658
Plaque Psoriasis	UC	1	57	Unknown	China	NCT03424629
Plaque Psoriasis	AT	NA	8	Enrolling by invitation	China	NCT04275024
Plaque Psoriasis	AT	1/2	16	Recruiting	China	NCT04785027
Plaque Psoriasis	AT	1/2	7	Active, not recruiting	China	NCT03265613
Plaque Psoriasis	AT	1/2	8	Enrolling by invitation	China	NCT03392311
Epidermolysis Bullosa	UCB	2	75	Recruiting	USA	NCT01033552
Epidermolysis Bullosa	UCB	2	84	Recruiting	USA	NCT02582775
Epidermolysis Bullosa	BM	1/2	10	Not yet recruiting	USA	NCT04173650
Diabetic Foot Ulcers	BM	1	12	Unknown	Israel	NCT01686139
Plaque Psoriasis	UCB	1	9	Recruiting	Korea, Republic of	NCT02918123
Atopic Dermatitis	AT	1/2	90	Enrolling by invitation	Korea, Republic of	NCT04725136
Atopic Dermatitis	AT	3	197	Completed	Korea, Republic of	NCT03269773
Diabetic Foot Ulcers	NA	1/2	51	Unknown	Colombia	NCT02943486

Adipose tissue (AT), Bone marrow (BM), Umbilical cord (UC), Umbilical cord blood (UCB)

In vivo reports have indicated that injection of green fluorescence protein (GFP) + allogeneic BM-MSCs could ameliorate wound healing in diabetic mice. Treated wounds displayed augmented wound closure, with improved re-epithelialization, cellularity, as well as angiogenesis. Remarkably, BM-MSCs expressed the keratinocyte-specific protein keratin and shaped glandular structures, signifying a straight influence of BM-MSCs on cutaneous renewal. Molecular analysis showed great levels of VEGF and angiopoietin-1 (Ang1) in BM-MSCs secretome, highlighting the importance of the MSCs-elicited angiogenesis in cutaneous wound healing [53]. Similarly, UC-MSCs labeled with 5-bromodeoxyuridine (BrdU) restored the healing of murine skin defect wounds, as well as differentiated into keratinocytes in the wound area [8]. MSCs also could promote the regeneration of dystrophic epidermolysis bullosa (DEB) wounds by stabilization of dermal and epidermal healing and improving skin integrity by de novo construction of functional immature anchoring fibrils. Further, anti-inflammatory effects elicited by MSCs could suppress immune cell infiltration into damaged DEB skin in the late phase of regeneration [54]. Besides, MSCs seeded on bioengineered scaffolds could improve skin wound healing in rats. In fact, implanted scaffold improves the quality of regenerated skin, promotes reepithelization, stimulates neo-angiogenesis, and also supported a better return of hair follicles and sebaceous glands by up-regulation of matrix metalloproteinase 9 expressions (MMP9) in the extracellular matrix (ECM) [55]. Also, Munir et al. found that injection of MSCs primed with the LPS resulted in better wound healing compared with the non-primed MSCs. Indeed, LPS-primed MSC induced toll-like receptor 4 (TLR4) pathways and thereby promoted neutrophil and macrophage recruitment to wound tissue, culminating wound healing [56]. On the other hand, there is clear evidence signifying that hyperbaric oxygen induces Wharton's Jelly (WJ)-MSCs proliferation, angiogenesis, and wound healing competencies in diabetic mice [57].

A myriad of studies has shown that MSCs delivery by scaffold could result in more desired wound healing activities in vivo than direct administration of MSCs. Meanwhile, MSCs delivery using collagen hybrid scaffolds promoted collagen deposition and also angiogenesis in diabetic wound healing in vivo [58]. Likewise, scaffolds comprising chitosan, adipic acid dihydrazide (ADH), and hyaluronic acid-aldehyde (HA-ALD) may serve a moist and inflammatory relief milieu to trigger MSCs proliferation or release of growth factors, thereby supporting wound healing. Indeed, constructed scaffold loaded with BM-MSCs could adjust the inflammatory milieu by suppressing the functions of M1 macrophages and conversely inducing M2 macrophages activities. These effects are closely linked to granulation tissue creation, collagen deposition, nucleated cell proliferation, neovascularization, and finally diabetic wound healing [59]. Too, MSCs delivery employing small intestinal submucosa (SIS) as a cell carrier may suppress the wound tissue’s inflammation and also accelerate the generation of skin regeneration-related growth factors [60].

## Current limitations of MSCs application in wound healing

In spite of advancements in MSC-based therapies, various challenges have hindered their widespread utility [61]. The existence of the considerable inconsistency in the delivery protocols, wound models, and MSCs populations among conducted studies make it difficult to define the influence of delivery time, delivery site, delivery systems, and also quantity of administered cells on MSCs engraftment outcomes [62, 63]. Different strategies for MSC delivery to cutaneous wounds are used: direct topical/spray, scaffold loaded, subcutaneous injection, or systemic delivery [64]. Thereby, MSCs' persistent engraftment seems to be modified by the delivery system of MSCs into the site of the wound. Donor immunogenicity and the wound milieu also are other factors determining the outcome of engraftment [64, 65]. Albeit, it remains to be comprehended whether persistent engraftment is universally required in wound healing. Besides, although there is some evidence validating the MSCs differentiation into mature and functional cutaneous cells, there are some other inconsistent reports [62]. These reports suggest that paracrine factors released from MSCs play a central role in the process of wound healing by plummeting wound inflammation and instigating tissue repair [66–68]. Furthermore, large-scale clinical trials are urgently prerequisites to address the potential therapeutic merits of MSCs in addition to evaluating MSC's possible transformation and tumor formation [69, 70]. Such comprehensive studies should be undertaken into consideration by a technical community concentrating on the practical MSC utility in wound healing to enable the optimal preparation of MSC-based products for more appreciated therapies.

## Exosomes biogenesis

The first reliable report indicating the presence of extracellular vesicles (EVs) (e.g., exosome) was published in 1946 [71]. In 1977, De Broe pronounced the secretion of some “membrane fragments” as a unique attribute of viable cells [72]. In the 1980s, these “fragments” were notified as platelet “dust” or cellular debris budding directly from the plasma membrane [73]. In 1983, Harding and Johnstone showed that transferrin receptors in association with small 50 nM vesicles were secreted from maturing blood reticulocytes into the extracellular space by receptor-mediated endocytosis and recycling [74, 75]. Rose Johnstone called these EVs “exosomes.” In the 1990s, exosomes were also validated to be secreted by B lymphocytes [76] and dendritic cells [77] via an alike mechanism.

**Fig. 2:**
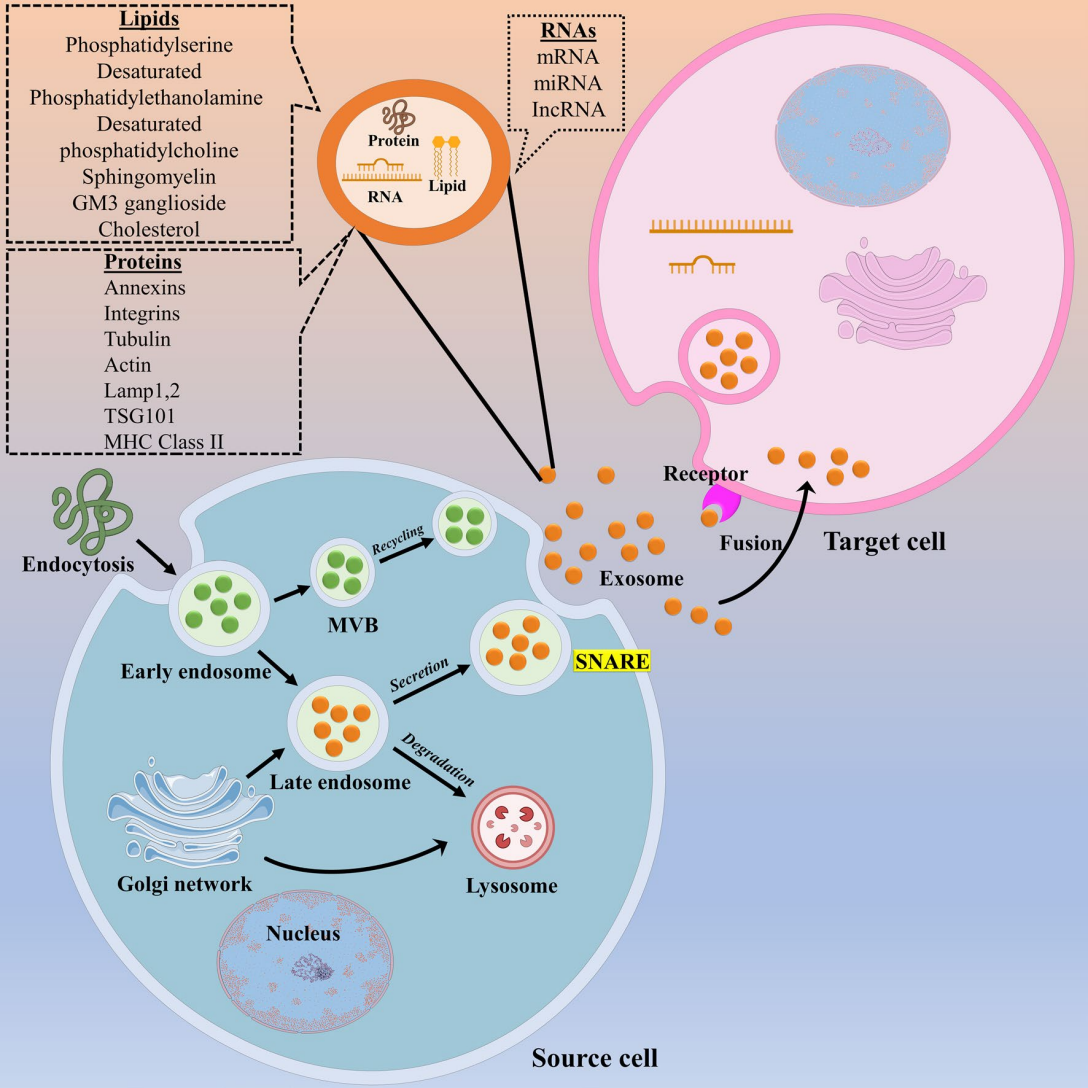
Schematic demonstration of the biogenesis, ingredients, and also secretion of exosomes. The exosomes are generated by the budding of the endocytic membrane and the creation of intraluminal vesicles (ILVs) inside the cell. During maturation, RNAs, proteins, and lipids are fused into ILV by endosomal complexes required for transport (ESCRT)‐dependent or ESCRT‐independent way, and early endosome maturation gives rise to multivesicular bodies (MVBs). The MVBs can be transferred to the trans-Golgi network (TGN) for endosome recycling, or to lysosomes for degradation, or incorporated with the plasma membrane through some dominant factors, such as Ras-related in the brain (Rab) GTPases and soluble NSF attachment protein receptor (SNARE) complexes. Upon MVB incorporation with the cellular membrane, exosome’s secretion into the extracellular space is completed, and ultimately secreted molecules are transported to recipient cells by endocytosis, direct membrane fusion, and receptor–ligand interaction

The biogenesis of exosome is a firmly regulated process and comprised of three main stages: (1) generation of endocytic vesicles by invagination of the plasma membrane, (2) generation of multivesicular bodies (MVBs) by inward budding of the endosomal membrane, and (3) incorporation of established MVBs with the plasma membrane and secretion of the vesicular contents, named exosomes (Fig. 2) [78]. Exosomes possess a lipid bilayer and also a small cytosol without any cellular organelles. Upon secretion, exosomes act as messengers, and thereby shape communication with other cells through the procedure of vesicular docking and incorporation with the assistance of soluble N-ethylmaleimide-sensitive factor attachment protein receptor (SNAREs) complexes and the endosomal sorting complex required for transport (ESCRT) [79, 80]. The ESCRT includes four dissimilar protein multiplexes, ESCRT-0, -I, -II, and -III, and the associated AAA ATPase Vps4 complex [81]. Importantly, any disruption in ESCRT-0 proteins, Hrs and TSG101, and the ESCRT-I protein, signal transducing adaptor molecule 1 (STAM1), could abrogate the release of exosomes. In contrast, the depletion of ESCRT-III and its accompanying proteins, CHMP4C, VPS4B, VTA1, and ALIX, could augment exosome release [81]. Also, the release of exosomes mainly improves in COS cell, fibroblast-like cell lines, following transfection with the integral membrane protein, SIMPLE, while deregulation in SIMPLE activity restricts exosomes biogenesis [82]. Nonetheless, there is strong proof showing that MVB biogenesis can be accomplished without ESCRTs, as evidenced by the formation of intraluminal vesicles (ILVs) in the lack of main subunits of all four ESCRT complexes. In addition, lipids (e.g., ceramide) contribute to vesicle biogenesis and transport procedures like membrane deformation, fission, and fusion [83]. Likewise, heat shock proteins, milk fat globule-EGF factor 8 protein (MFG-E8 or lactadherin), GTPases, platelet-derived growth factor receptors (PDGFR), annexins, flotillins, and also tetraspanins mostly participate in exosome biogenesis [84]. Moreover, v-SNAREs and t-SNAREs involve in the facilitating anterograde and retrograde protein-sorting stages between the Golgi and the plasma membrane [84].

Exosomes are mainly consisting of functional proteins, mRNA, and microRNA. They comprise proteins from endosomes, the plasma membrane, and the cytosol; however, some compositions from the nucleus, mitochondria, endoplasmic reticulum, and Golgi apparatus can be found in exosomes [85]. Universally, the protein compositions of exosomes rely on their origin cell. Some biomarkers like tumor susceptibility gene 101 (TSG101), charged multivesicular body protein 2a (CHMP2A), Ras-related protein Rab-11B (RAB11B) in association with CD9, CD63, as well as CD81 proteins are chiefly used for exosome characterizing [84, 86, 87]. The exosome macromolecular ingredients have a dominant role in cellular activities and pathological conditions, including inflammation, immunological reactions, angiogenesis, cell death, neurodegenerative diseases, and also tumors [88–90].

## Improving the yields and functionality of MSCs-derived exosome

According to the literature, there exist various challenges in the clinical use of exosomes, particularly in regenerative medicine. Among multiple suggested isolation and purification plans, there is no universal method for the separation of exosomes from other micro-particles [91, 92]. To date, several established approaches, such as differential ultracentrifugation, density gradients, precipitation, filtration, and size exclusion chromatography have successfully been applied for the isolation of exosomes from origin cells (e.g., MSCs) [93]. Nevertheless, the limited release of exosomes from origin cells barricades their large-scale manufacture and thereby abrogates their medical use. Upon a few passages, MSCs enter replicative senescence, and so their inherent potential to secret vesicles is impaired. Hence, finding or designing approaches or molecules for circumventing the limited quantities of secreted exosomes are critically required. Some reports have shown that the tangential flow filtration (TFF) system-based method could offer higher quantities of exosomes derived from UC-MSCs than the ultracentrifuge (UC)-based conventional method [94]. Another study revealed that ultrasonication of ultracentrifuged MSC-exosome followed by consistent centrifugation and filtration enables increasing the exosome yield by 20-fold [95]. A hollow fiber three-dimensional (3D) culture system also could serve as a proficient approach for the incessant manufacture of MSC-exosome [96, 97]. As well, expansion of UC-MSCs in 3D cultures combined with the conventional differential UC resulted in 20-fold more exosomes than two-dimensional (2D) cultures combined with the conventional differential. Importantly, TFF combined with 3D MSCs cultivation potently increased the yield of exosomes (3D-TFF-exosomes) up to sevenfold over 3D-UC-exosomes [98]. Likewise, biomaterial 45S5 Bioglass^®^ (BG) could significantly improve MSCs-derived exosomes yields by inducing the expression of neutral sphingomyelinase-2 (nSMase2) and Rab27a, eliciting nSMases and Rab GTPases axes and ultimately augmenting exosome secretion [99, 100]. Alginate hydrogel also could facilitate the secretion of utmost levels of cytokines and growth factors, like fibroblast growth factor 2 (FGF-2), insulin-like growth factor (IGF), hepatocyte growth factor (HGF), and leukemia inhibitory factor (LIF) from seeded MSCs than normal MSCs [99]. Further, Avitene Ultrafoam collagen hemostat also could lead to the releases of higher quantities of exosomes by BM-MSCs, as shown in brain traumatic mice models [101].

Preconditioning of human MSCs with varied molecules, such as lithium could restore the neuroprotective capabilities of MSCs arbitrated by improved secretion of exosomes [102]. Similarly, the secretion of exosomes can be increased by preconditioning with cytochalasin B or antifungal reagents nitrefazole [103]. Likewise, exosome derived from MSCs preconditioned with interferon (IFN)-γ, tumor necrosis factor α (TNFα), IL-1β, IL-6, and TGF-β may offer better therapeutic benefits in vivo [104–106]. Similarly, exosomes derived from MSCs preconditioned with hypoxia could show improved angiogenesis compared with the exosomes derived from MSCs cultivated under normoxic conditions due largely to the induction of VEGF signaling pathways in hypoxia-preconditioned MSCs [107, 108]. As well, overexpression of several genes such as GATA binding protein 4 (GATA4), platelet-derived growth factor D (PDGF-D), miR-126, miR-133b, miR-119a, miR-92a-3p, miR-181-5p, miR-20a, miR-30d-5p, tumor necrosis factor-related apoptosis-inducing ligand (TRAIL), and hypoxia-inducible factor-1 alpha (HIF-1α) in human MSCs could robustly ameliorate the therapeutic merits of MSCs-exosome [103, 109–114].

## MSCs role in wound healing

### Immunomodulation

In 2000, Liechty and coworkers for the first time indicated that MSCs possess unique immunological competencies, permitting them to modify the immunological reactions [115]. Thereby, it was suggested that MSCs therapy could be an efficient strategy for attenuation of inflammation and so improving wounds healing [116]. Universally, MSCs can moderate immunological response through a diversity of processes, including inhibition of T-cell activities along with the progression of macrophages shift from M1 to M2 (34). MSCs could also shape the cytokine-producing profile of naive and effector T cells, natural killer (NK) cells, and also dendritic cells (DC) cells to stimulate anti-inflammatory or tolerant phenotypes. MSCs function largely mediated through cell-to-cell contact accompanied by the secretion of a diversity of factors. MSCs-elicited immune-modulatory effects mainly rely on the TGF-β1, PGE2, hepatocyte growth factor (HGF), indoleamine-pyrrole 2,3-dioxygenase (IDO), NO, IL-4, and IL-10 [117–120]. These factors suppress the expression of pro-inflammatory cytokines like IFN-γ, TNF-α, IL-1, and IL-6, and conversely stimulate expression of IL-4, and IL-10 secretion mainly by T regulatory and M2 cell [121], and so untimely prepare a solid milieu to tissue repair. Apart from their application in wound healing, MSCs therapy could cause continued skin graft survival through inhibition of lymphocyte reactivity in vivo [122].

A study has shown that carrier-delivered Ad-MSCs could attenuate TNF-α-mediated inflammation, and also improve anti-inflammatory M2 macrophage quantities, and so accelerate full-thickness cutaneous wound healing [51]. Likewise, UC-MSC could also migrate into the wound area and robustly diminish the quantities of infiltrated inflammatory cells and IL-1, IL-6, and TNF-α level in wound tissue. In contrast, they could boost the levels of IL-10 and TNF stimulated gene-6 (TSG-6) in severely burned rats [123]. The MSC-secreted TSG-6 has been recognized to induce wound healing by limiting macrophage activation, inflammation, and also fibrosis [124]. Furthermore, TSG-6 released by UC-MSCs mitigated severe burn-induced excessive inflammation through the down-regulation of activations of P38 and JNK signaling [125]. Meanwhile, a study in rats showed that UC-MSCs could only down-regulate P38 and c-Jun N-terminal kinases (JNKs) signaling, but did not affect extracellular signal-regulated kinase (ERK) activation in peritoneal macrophages of severe burn rats [125]. Additionally, suppression of P38 and JNK activations significantly reduced the excessive inflammation induced by severe burn. On the other hand, TSG-6 in MSCs robustly stimulated phosphorylation of P38 and JNK signaling and thereby lessened MSCs-mediated therapeutic effect on excessive inflammation [125]. Similarly, human gingiva-derived MSCs (GMSCs) induced polarization of M2 macrophages and accelerated cutaneous wound healing in vivo [126]. In vitro, GMSCs supported the acquirement of anti-inflammatory M2 phenotype in macrophage in co-culture condition evidenced by an enhanced expression of mannose receptor (MR; CD206) and IL-10 secretion, and also by the inhibited generation of TNF-α [126]. In vivo, GMSCs injection by intravenous route resulted in M2 polarization, and also alleviated local inflammation through suppressing the infiltration of inflammatory cells and generation of IL-6 and TNF-α accompanied by elevated expression of IL-10. It was found that reduction in TNF-α secretion by macrophages might arise from impairment in nuclear factor-κB (NF-κB) p50 activation induced by GMSCs [126]. As well, evaluation of MSCs involvement in wound healing by employing small intestinal submucosa (SIS) as a cell carrier revealed that stem cells could modify inflammatory responses in wound tissue as well as augment the skin regeneration-related growth factors, thereby facilitating skin recovery in nude mice [60].

### Angiogenesis

As multipotent progenitors, MSCs have demonstrated their great competence to differentiate into several cell types and could trigger endogenous angiogenesis by microenvironmental modulation. They can induce activation of STAT3, and consequently boost proliferation and migration of endothelial cells (ECs) by secreting HGF, IL-6, b-FGF, and VEGF [127]. As well, MSCs could be induced to produce other pro-angiogenic factors, involving angiogenin, Ang1, activin A, MMP-9, granulocyte–macrophage colony-stimulating factor (GM-CSF), and urokinase-type plasminogen activator, making them an ideal alternative for stimulation of vascularization [128]. In this regard, a clinical trial in participants with critical limb ischemia established by diabetes showed that MSCs therapy resulted robustly in alleviation in ulcer size, pain-free walking time, and percutaneous tissue oxygen. Moreover, the MSCs group showed greater levels of VEGF and FGF-2 than the control group. These findings delivered the proof of the theory that MSCs could hold great promise for vascular regeneration as well as wound healing [129]. Another study in severely burned rats revealed that UC-MSC therapy could prompt neovascularization by restoring VEGF levels in wounds tissue [123]. Importantly, the ratio of collagen types I and III in the UC-MSC group was noticeably higher compared with the control group [123]. As well, GFP + allogeneic BM-MSCs prominently accelerated wound healing in normal and diabetic mice in comparison to the allogeneic neonatal dermal fibroblasts [53]. Analyses verified the engraftments of 27% of administrated allogeneic BM-MSCs at 1-week post-administration. The treated wound tissue exhibited ameliorated wound closure, with better re-epithelialization, cellularity, as well as angiogenesis. Importantly, BM-MSC-conditioned medium stimulated endothelial cell tube formation in vitro because of the existing high levels of VEGF and Ang1 [53]. The Ang-1 produced by human MSCs can trigger vessel remodeling and maturation by inducing the receptor tyrosine kinase Tie2, and so its existence in wound tissue in association with other pro-angiogenic factors can result ultimately in neovascularization [130, 131]. Besides, MSCs preconditioned with IFN-γ and TNF-α showed robust potential to induce angiogenesis, facilitate collagen deposition, and accelerate wound closure mainly by secreting VEGFC, but not VEGFA [132]. In this regard, secretion of PDGFR-β has been outlined as another mechanism applied by MSCs to ameliorate wound injuries. Correspondingly, PDGFR-β-expressing MSCs could produce higher levels of Ang1, Ang2, VEGF, b-FGF, and PDGF than PDGFR-β negative MSCs. In vivo, PDGFR-β-positive MSCs showed prominent competence for incorporation into the wound tissue, efficient engraftment, and also sustained wound healing mediated chiefly by enhanced angiogenesis [133]. As well, MSC-seeded hydrogels demonstrated potent capability to stimulate wound healing with augmented levels of VEGF and other pro-angiogenic cytokines within the wounds, indicating that biomimetic hydrogels scaffold could prepare a functional niche to support MSC regenerative potential and improve wound healing [134]. Interestingly, evaluation of possible effects of the SDF-1α gene-activated collagen-based scaffolds on MSCs driven angiogenesis signified that collagen-based scaffolds combined with SDF-1α gene therapy could lead to the boosted pro-angiogenic response, proposing a capable tactic to circumvent poor vasculature during wound healing [135]. Since SDF‑1 treatment could improve endothelial progenitor cells (EPCs) proliferation, migration, and tube formation through the multiple mechanisms, such as activating AKT and ERK signaling axes, it appears that a special focus on its role in wound healing procedure is of utmost importance to offer more effective therapeutic modalities [136, 137].

### Differentiation

As defined, MSCs are universally noticed as self‐renewable, multipotent progenitor adult stem cells, which are found in BM, peripheral blood, skin, etc. [138]. This wide differentiation potential, make them an attractive and rapidly evolving option for acute and chronic wounds treatment. For instance, BM-MSCs cultivation on electrospun nanofibers of collagen and poly (l-lactic acid-co-e-caprolactone) (PLLCL) could support their fibroblastic differentiation [139]. Intriguingly, there are some reports, which show that MSC could migrate in vivo to the injured site and then differentiate into multiple skin cell types such as keratinocytes and thereby could support wound healing [140, 141].

Assessment of the therapeutic advantages of BM-MSCs therapy in wound healing applying an excisional wound splinting model demonstrated that intervention considerably accelerated wound healing in diabetic mice [53]. Importantly, BM-MSCs expressed the keratinocyte-specific protein keratin and shaped glandular structures, demonstrating straight participation of BM-MSCs in cutaneous regeneration [53]. In addition to the direct differentiation into keratinocytes, MSCs could express EGF, FGF-2, and keratinocyte growth factor (KGF), and so supports early stimulation of keratinocyte migration and function in wound tissue [142]. Moreover, local application of UCB-MSCs labeled with BrdU caused the healing of mice skin defect wounds along with their direct differentiation into keratinocyte in the wound tissue [8]. So, other studies exhibited that Ad-MSC could transdifferentiate into keratinocyte-like cells following co-culture with human keratinocytes or with conditioned medium derived from cultured human keratinocytes (KCM) and also could engineer a stratified epidermis. The Ad-MSC-derived keratinocytes expressed specific keratinocyte markers such as cytokeratin-5, involucrin, filaggrin, and stratifin, and thus could be employed to ameliorate cutaneous wounds [143]. As well, nanofibers comprising polyvinyl alcohol (PVA), gelatin, and azide also promoted the Ad-MSCs differentiation into keratinocytes [144]. In another study, Mishra and his coworkers showed that the co-culture of human MSCs with KCM could stimulate dermal myofibroblast-like differentiation of human MSCS, as evidenced by detecting cytoskeletal markers and also up-regulated expression of SDF-1, CXCL5, IL-8, and IL-6 in vitro [145]. In vivo, they found the dermal myofibroblast-like human MSCs sustained the wound healing process by secreting soluble factors [145]. On the other hand, Lee et al. described that efficient fibroblastic differentiation of human MSCs could be achieved by applying connective tissue growth factor (CTGF) as well as ascorbic acids [146]. Other research teams successfully conducted fibroblasts differentiation from human embryonic stem cell (ESC)-derived MSCs [147]. The achieved fibroblasts presented a substantial enchantment in the expression of type I and III collagen, fibronectin, and fibroblast-specific protein-1 (FSP-1). Robustly, administration of these ESC-MSC-fibroblast caused wound tissue regeneration in a pressure ulcer mice model, offering capable treatments for skin ulcers therapy [147]. Apart from keratinocyte and fibroblast, other studies have demonstrated MSCs differentiation into ECs and pericytes, representative of a capability for neovascularization [148]. The pericytes elongate around ECs and are functionally accompanying with modifying vessel stabilization, and therefore act as prominent cells during vasculogenesis and angiogenesis [149].

## MSC-derived exosome in chronic wound healing

The MSCs differentiation is contributed to the wound healing procedure; however, it seems that MSCs other attributes such as suppressing inflammation, anti-fibrotic activities, and also inducing angiogenesis largely mediated by soluble factors (e.g., cytokines, growth factors, chemokines, mRNA, proteins, and microRNAs) play the more prominent role than direct differentiation during wound healing upon MSCs therapy. Thereby, utilizing MSC-derived exosome has attracted great attention to accelerate cutaneous wound healing (Table 3). Concerning the recent reports, human-induced pluripotent stem cell (hiPSC)-MSC-derived exosome could trigger the proliferation and migration of human dermal fibroblasts (HDFs) and human umbilical vein endothelial cells (HUVECs) in vitro in addition to potentiating the expression of type I, III collagen and elastin in HDFs [150]. Remarkably, MSCs-derived exosomes facilitate MSCs-mediated hindrance of the inflammatory microenvironment and also pro-angiogenesis of ischemic tissue of diabetic foot ulcers (DFU). Meanwhile, exosomes achieved from MSCs using ultracentrifugation have shown a great level of the pro-angiogenic miRNA, miRNA-21-5p. Molecular analysis by knockdown and overexpression of miRNA-21-5p by manipulating MSCs has validated the biological functions of exosome miRNA-21-5p, such as in vitro cell proliferation along with in vivo pro-angiogenesis in DFU rat models. Further, miRNA-21-5p could improve angiogenesis by stimulation of VEGFR and also activations of serine/threonine kinase AKT and mitogen-activated protein kinase (MAPK) in recipient cells (e.g., ECs), facilitating ischemic tissue repair and angiogenesis in DFU models [151]. Moreover, exosomes derived from MSCs stimulate macrophage polarization, while depletion of MSC’s exosome diminishes the macrophage’s M2 phenotype. Importantly, MSCs administration without exosomes results in the lower frequency of M2 macrophages at the wound site concurrently delayed wound healing compared to the exosome therapy. Investigations have shown that miR-223, derived from MSCs-exosome, plays a central role in the adjustment of macrophage polarization upon targeting PBX/Knotted 1 homeobox 1 (pknox1), indicating that wound healing could be supported through conveying the exosome-derived miRNAs [152]. There is another report showing that exosome-delivered Wnt4 proposes new facets for the therapeutic approach of MSCs in cutaneous wound healing. The Wnt4 improves β-catenin nuclear translocation and functions to stimulate proliferation and migration of skin cells, enabling wound re-epithelialization and cell proliferation [153]. Besides, fetal dermal MSCs (FDMSCs)-derived exosomes sustained wound healing process in a mouse full-thickness skin wound model by induced proliferation, migration, and secretion of resident fibroblast. It seems that FDMSC-exosomes elicit wound healing by inducing fibroblast cell motility and secretion potential by the Notch signaling pathway [154]. Moreover, inhibition of the TGF-β/Smad signal pathway [155], activation of the NF-κB [156], and also hindrance of the apoptosis-inducing factor (AIF) translocation [157] are other suggested mechanisms applied by MSCs-exosomes to accelerate wound healing in vivo.

**Table 3:** Mesenchymal stem/stromal cell (MSC)-derived exosome therapies for cutaneous wound healing

Cell source	Model	Results	References
BM	In vivo	Inducing the macrophage polarization by MSCs-exosome due to the existence of miR-223, supporting PBX/Knotted 1 homeobox 1(pknox1) targeting	[208]
UC	In vivo	Stimulating the wound re-epithelialization and cell proliferation by inducing Wnt/β-catenin through the UC-MSCs-exosome	[153]
iPSCs-MSC	In vivo	Enabling cutaneous wound healing by improving collagen synthesis as well as inducing by human-induced pluripotent stem cells- MSCs-exosome	[150]
BM	In vivo	Accelerating wound healing in DFU mice by MSC-exosomes overexpressing lncRNA H19	[165]
BM	In vitro In vivo	Inhibition of the expression of IL-1β, TNF-α, and iNOS, and augmenting the expression of anti-inflammatory factor IL-10 in vitro by melatonin-preconditioned MSC-exosomes Amelioration of the diabetic wound healing by adjusting the macrophage M1 and M2 polarization by regulation of the PTEN/AKT pathway through melatonin-stimulated MSC-derived exosomes in vivo	[162]
BM	In vitro	Improving the endothelial cells (ECs) proliferation, and angiogenesis through regulating AKT/eNOS pathway by MSCs-exosome in vitro	[209]
UC	In vitro	Suppressing myofibroblast differentiation through suppressing the TGF-β/SMAD2 pathway by UC-MSCs-exosome	[210]
BM AT UC	In vitro	Verifying the presence of VEGFA, FGF-2, HGF, and PDGF-BB in exosomes derived from BM, AT, and UC	[211]
iPSCs-MSC	In vitro	Stimulating the human keratinocytes (HaCaT) and human dermal fibroblasts (HDFs) proliferation by iPSC-MSC-exosomes	[212]
BM	In vitro	Inducing the proliferation and migration of fibroblasts, and stimulating angiogenesis in vitro by activating Akt, ERK, and STAT3 axes, and also improving the expression of an HGF, IGF1, NGF, and SDF1	[127]
UC	In vitro	Facilitating the collagen I and elastin synthesis in vitro by UC-MSCs-exosome	[213]
AT	In vitro	Triggering the endothelial cell angiogenesis by transferring miR-125a by MSCs-exosome	[214]
WJ	In vivo	Inhibition of skin cell death via inhibiting the AIF nucleus translocation and accelerating cutaneous wound healing by MSC-exosomes	[157]
BM	In vivo	Amelioration of scar pathological injury, and reducing the inflammatory molecular generation in vivo by MSC-exosomes overexpressing TSG-6	[166]
BM	In vitro	Stimulating the in vitro wound healing by targeting the biological features of skin keratinocytes and fibroblasts as well as eliciting the angiogenesis by MSC-exosomes	[16]
UC	In vivo	Inducing the regenerative wound healing by inhibiting the TGF-β receptor by UC-MSCs-exosome	[215]

Induced pluripotent stem cell (iPSC), Adipose tissue (AT), Bone marrow (BM), Umbilical cord blood (UCB), Wharton's jelly (WJ), Diabetic foot ulcer (DFU), Inducible nitric oxide synthase (iNOS), Phosphatase and tensin homolog (PTEN), Endothelial NOS (eNOS), Fibroblast growth factors (FGFs), Hepatocyte growth factor (HGF), Platelet-derived growth factor (PDGF), Transforming growth factor-beta (TGF-β), Extracellular signal-regulated kinase (ERK), Vascular endothelial growth factor (VEGF), MicroRNAs (miRNAs), Signal transducer and activator of transcription 3 (STAT3), Insulin-like growth factor (IGF), Nerve growth factor (NGF), Stromal cell-derived factor 1 (SDF1)

Today, some strategies have been developed to achieve more festive MSCs-derived exosomes for supporting more desired outcomes. These strategies include MSCs pretreatment, MSCs genetically modification, and also exosome delivery using hydrogel.

### Preconditioned MSCs-derived exosome

Current studies have displayed that activation of MSCs using erythropoietin (EPO) as an anti-inflammatory, pro-angiogenic cytokine ameliorates the effective healing of DFU. Indeed, EPO induces MSCs to produce VEGF, diminishes the damage to MSCs, supports MSCs proliferation and migration capabilities, and finally obstruct secretion of TNF-ɑ from MSCs in vitro. Exosomes derived from EPO-activated MSCs trigger the angiogenesis process and also inhibit monocyte activities in vivo in wound tissue in DFU [158]. Similarly, exosomes derived from BM-MSCs preconditioned with atorvastatin (ATV) demonstrated prominent pro-angiogenic competence in diabetic wound healing. Studies in full-thickness skin defects in streptozotocin (STZ)-induced diabetic rats confirmed the potential of exosomes derived from ATV-MSCs to support the wound regeneration by promoting the formation of blood vessels than exosomes derived from non-preconditioned MSCs. In vitro, these exosomes heightened the proliferation, migration, tube formation, and VEGF level of ECs. Molecular investigations revealed that the observed desired effects might be attributable to activation of AKT/ endothelial nitric oxide synthase (eNOS) pathways and also up-regulation of miR-221-3p in ECs by exosomes derived from ATV-MSCs [159]. It has previously been found that miR-221 is an influential player in vascular biology by stimulating its impacts on vascular smooth muscle cells (VSMCs) and ECs, and thereby any modification in its expression levels by MSCs-derived exosome can affect the biological activities of ECs and so target angiogenesis in injured tissue [160]. Likewise, exosomes derived from MSCs preconditioned with pioglitazone enhanced the cell viability and proliferation of HUVECs in DFU model [161]. Moreover, exosomes elevated the biological activities of HUVECs, involving migration, tube formation, wound healing, and VEGF expression in vitro. Moreover, exosomes augmented the protein expression of p-AKT, p-PI3K, and p-eNOS and conversely inhibited phosphatase and tensin homolog (PTEN), culminating cells viability and proliferation. More importantly, pioglitazone preconditioned MSCs-derived exosomes supported diabetic wound healing by improved enhanced angiogenesis, collagen deposition, ECM remodeling, VEGF, and also CD31 expression. These findings offer the evidence of ideas that the PI3K/AKT/eNOS pathway is one of the key singling axes affected by MSCs-exosome to mediate therapeutic effects in recipient cells [161]. Furthermore, melatonin (MT) [162] or deferoxamine [163]-pretreated MSCs-derived exosomes provoked diabetic wound healing by inhibiting the inflammatory response mediated by enhancement of the ratio of M2 polarization to M1 polarization by inducing the PTEN/AKT signaling pathway.

### Modified MSCs-derived exosome

Irrespective of pretreatment, it has been presented that overexpression of some genes in MSCs could potently promote the therapeutic advantages of their exosomes. For instance, exosomes from Ad-MSCs overexpressing nuclear factor erythroid 2-related factor 2 (Nrf2) improved granulation tissue creation, angiogenesis, and levels of growth factor expression, while attenuated levels of inflammation and oxidative stress-related proteins in wound beds, as shown in the DFU model [164]. As well, the existence of the long noncoding RNA (lncRNA) H19 in MSC-derived exosomes overexpressing lncRNA H19 was found that are involved in accelerating wound healing in DFU through targeting PTEN expression in fibroblasts. Mechanistically, PTEN down-regulation led eventually in the stimulating the PI3K/AKT1 signaling axis, which in turn, sustained wound healing in DFU possibly by potentiating the fibroblast proliferation and migration, as well as suppressing apoptosis and inflammation [165]. Likewise, MSC-derived exosomes overexpressing TSG-6 could inhibit scar formation by decreasing inflammation and suppressing collagen deposition [166]. Further, studies have shown the HIF-1α [167], SDF1 [168], migration inhibitory factor (MIF) [169], and miR-30b [170] overexpression in MSCs-derived exosomes could exhibit superiority over normal exosome in terms of the pro-angiogenic activity and tissue repair capacity in vivo.

### MSCs-derived exosome’s delivery using hydrogel

Among the diverse kinds of biomaterials in which exosome infusion is employed, hydrogels have been confirmed to be the most user-friendly, economical, and accessible material. Thereby, hydrogels have been extensively utilized as a carrier for the continued and efficient delivery of exosomes [171]. Meanwhile, constructed combination of human UC-MSCs-derived exosomes and Pluronic F-127 (PF-127) hydrogel accelerated wound healing. In fact, topically used human UC-MSCs-exosome encapsulated in a thermosensitive PF-127 hydrogel led to a robust improved wound closure rate, boosted expression of CD31 and Ki67, augmented regeneration of granulation tissue, and raised expression of VEGF and TGFβ-1. These observations signify that biomaterial-based exosome therapy can serve an innovative therapeutic plan for the cutaneous regeneration of chronic wounds [172]. Also, chitosan-based hydrogel enriched with MSCs-exosome could enhance fibroblast cell migration and proliferation in vitro and also promote re-epithelialization in vivo [173]. Likewise, incorporation of gingival MSCs-derived exosomes to chitosan/silk hydrogel could efficiently support healing of diabetic skin defects by induction of the re-epithelialization, deposition, and remodeling of collagen and by stimulating angiogenesis and neuronal ingrowth [174]. Similarly, UC-MSCs-exosome loaded genipin crosslinked hydrogel enabled full-thickness cutaneous wound healing in rat animal models. These exosomes potently amended wound closure, improved re-epithelialization rates, as well as reinforced collagen deposition in the wound tissue [175]. In another study, Hu et al. used an extrusion-based cryogenic 3D printing technology to shape decellularized small intestinal submucosa (SIS) combined with mesoporous bioactive glass (MBG) and exosomes to produce a 3D scaffold dressing (SIS/MBG@Exos), permitting the prolonged secretion of bioactive exosomes [176]. The SIS/MBG@Exos hydrogel scaffolds enhanced the proliferation, migration, and angiogenesis of HUVECs in vitro, and also promoted diabetic wound healing by enhancing the blood flow of wounds and eliciting the angiogenesis process of the diabetic wound in vivo. The constructed scaffolds also induced the establishment of granulation tissue, collagen fiber deposition, and finally development of functional new blood vessels [176].

## Platelet-rich plasma (PRP) and biomaterials for wound healing

In addition to the MSCs-based therapies, other strategies based on biological products have been evolved to ameliorate skin wound healing. PRP has been attracted great attention because of its marked competence to induce and accelerate the wound healing process. Such events rely on the presence of a wide spectrum of cytokines and growth factors forming PRP, including PDGF, EGF, FGF, IGF1, IGF2, VEGF, TGF-β, and also keratinocyte growth factor (KGF) [177, 178]. Studies in full-thickness wounds C57/BL6 mice models signified that PRP could strongly ameliorate skin wound healing, by adjusting local inflammation along with improving angiogenesis and re-epithelialization largely mediated by enhanced secretion of IGF-1 and VEGF [179]. PRP treatment could also attenuate the production of inflammatory cytokines IL-17A and IL-1β [179]. PRP treatment also ameliorates the migration and proliferation of primary cultured ESCs, and also enhances their differentiation into adult cells following the modifying CD49f and keratin 10 and keratin 14 in vivo [179]. Moreover, a clinical trial on 24 patients with non-healing ulcers of different etiologies showed that PRP administration resulted in wound healing with a decrease in wound size (NCT03026855) [180]. On the other hand, a bio-functionalized scaffold comprising PRP and hyaluronic acid (HA) supported re-epithelialization and also showed more prominent regenerative potential in terms of epidermal proliferation and dermal renewal compared with HA alone in 182 patients affected by chronic ulcers [181]. As well, PRP plus HA could reduce ulcer area without any symptoms of infection in patients with pressure ulcers [182]. Apart from wound healing, PRP has demonstrated remarkable potential to stimulate hair regeneration by increasing terminal hair density/diameter in patients with androgenetic alopecia (AGA), a common form of hair loss [183, 184]. Another trials on 23 patients showed that PRP therapy led to clinical improvement in the mean number of hairs associated with no side effects [185]. Intervention caused an increase in Ki67 + keratinocytes in the epidermis and of hair follicular bulge cells, suggesting that PRP may serve as a safe and effective therapeutic approach against hair loss [185]. Moreover, PRP and also micrografts containing human follicle mesenchymal stem cells (HF-MSCs) enhanced hair growth by cellular proliferation to extend the anagen phase (FGF-7), convincing cell growth (ERK activation), motivating hair follicle development (β-catenin), and inhibition of apoptotic cues (Bcl-2 release and Akt activation) in patients with AGA [186]. As well, there is clear evidence indicating that other biomaterials regardless of HA, such as collagen hydrogels, dextran-based hydrogel, poly-thioketal urethane-based scaffolds, and copper-doped borate bioactive glass microfibers may entice wound healing in vivo [187].

## Conclusion

**Fig. 3:**
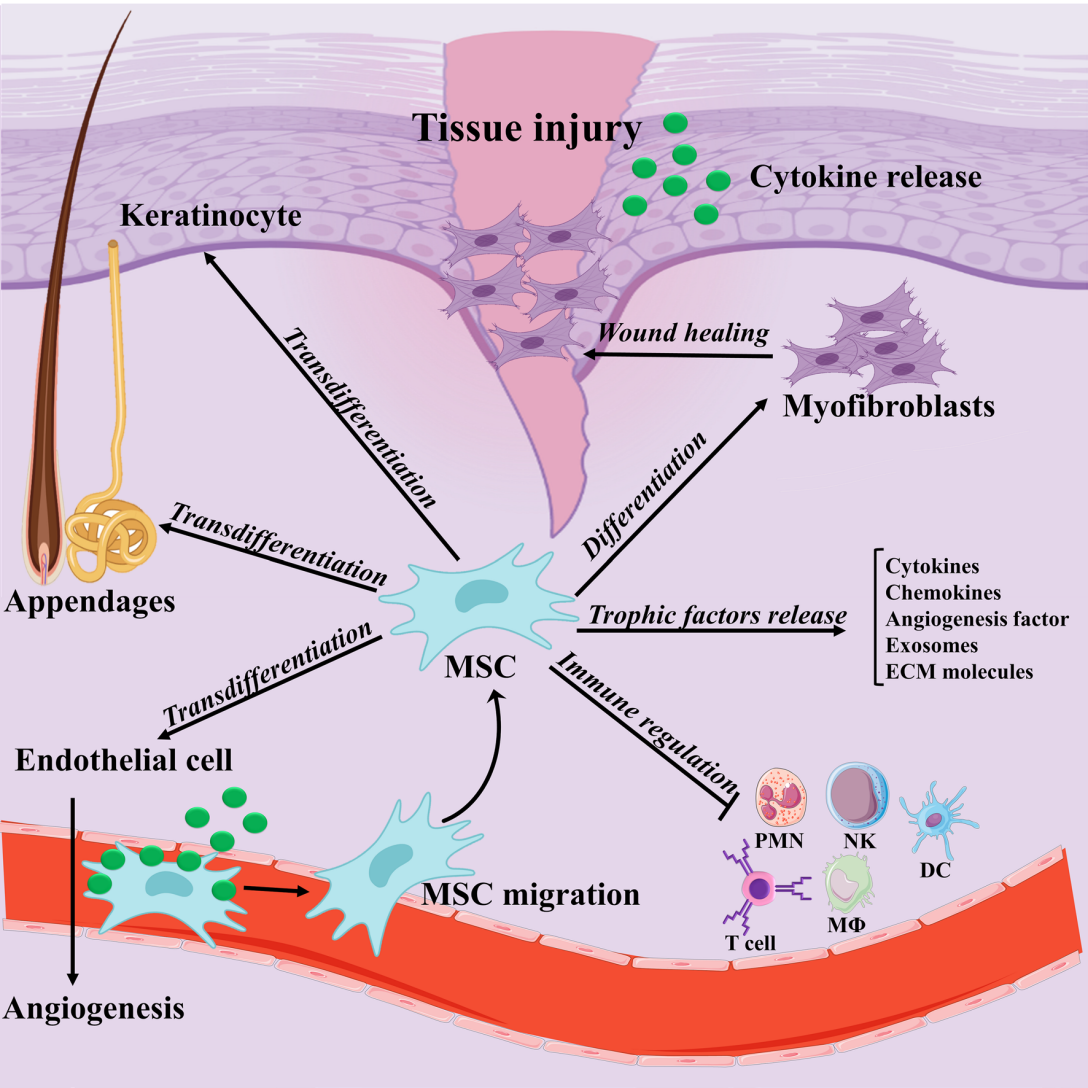
The suggested mechanisms of mesenchymal stem/stromal cells (MSCs)-mediated cutaneous wound healing. As demonstrated, accelerating cutaneous wound following MSCs injection via various routes (e.g., systemic injection) may be attributable to MSCs specific properties, such as inhibition of inflammation, secretion of a variety of soluble mediators, facilitating endothelial cell proliferation and also recruitment to wound tissue, transdifferentiation into skin-resident cells, and finally supporting matrix remodeling

During the last two decades, MSCs-based therapies because of their unique immunomodulatory attribute accompanied by their potential to secret pro-angiogenic factors and facilitate endothelial cell recruitment to wound tissue concomitant with their inherent capacity to establish skin-resident cells, and also accelerating matrix remodeling offer a paradigm shift in cutaneous wound healings (Fig. 3) [53, 188]. Nonetheless, the therapeutic competencies of MSCs in cutaneous wounds are presently only validated by some clinical studies with small sample sizes, short follow-up time, and the absence of randomized controlled trials. Notwithstanding, there are various trials presently recruiting participants to address the prolonged consequence of MSCs-based therapies on diabetic and venous ulcers. In sum, conduction of more comprehensive investigations into how MSC-elicited signals affect target cells and their cellular responses in vivo is of paramount importance [52]. Besides, MSCs-derived exosomes as nanoscale and closed membrane vesicles possess the capability to adjust various biological events related to the wound healing process, including cell proliferation, cell migration, and blood vessel formation [189]. The exosomes enable cell-free treatment, and thereby circumvent the safety issues regarding the application of the viable cells [190–192]. However, the application of MSC-exosome in the clinic is currently limited by lacking universally accepted standard of operation (SOP) for cell culture conditions and procedure, exosome separation and storage, optimum therapeutic dose and administration schedule, and also trustworthy potency tests to address the efficacy of exosome-based therapies [192]. Taken together, we guess that execution of large-scale studies based on MSCs and their exosome therapy can result in more desired outcomes in clinic.

## Data Availability

Not applicable.

## Abbreviation

MSCs: Mesenchymal stem/stromal cells
ECM: Extracellular matrix
VEGF: Vascular endothelial growth factor
PI3K: Phosphoinositide 3-kinase
BM: Bone marrow
UC: Umbilical cord
AT: Adipose tissue
DEB: Dystrophic epidermolysis bullosa
DFU: Diabetic foot ulcer
EVs: Extracellular vesicles
ECs: Endothelial cells
miRs: MicroRNAs
HUVECs: Human umbilical vein endothelial cells
TGFβ: Transforming growth factor β
